# Bioelectric Nerve Stimulation for Final Impression and Cameo Surface Recording for Completely Edentulous Patients: A Report of Two Cases

**DOI:** 10.7759/cureus.70689

**Published:** 2024-10-02

**Authors:** Sapna Rani, Vidushi Saxena, Pankaj Dhawan, Suraj Naidu

**Affiliations:** 1 Department of Prosthodontics, Manav Rachna Dental College, Faridabad, IND; 2 Department of Prosthodontics and Implantology, Manav Rachna Dental College, Faridabad, IND

**Keywords:** border molding, cameo surface, complete denture, completely edentulous patients, tens

## Abstract

Attaining the appropriate relation of the denture base with the tissues for optimum retention and support poses various challenges. Retention and support of a prosthesis depend on capturing mucosa and limiting structures in undistorted form. The impression in the geriatric population and patients with maxillary and mandibular defects poses a challenge for prosthodontists. An alternate impression-making technique, including transcutaneous electric nerve stimulation (TENS), can be viable for these clinical cases. In the TENS technique, electrodes are placed on the region of the posterior triangle of the neck and the pre-auricular region simultaneously on both sides to instigate muscle contractions in the target area. An impression is obtained in physiological harmony with surrounding musculature after applying TENS. TENS can induce muscle contractions when applied <10 Hz, while >20 Hz can cause paraesthesia. This case report presents two cases rehabilitated with complete dentures in which ultra-low frequency (ULF) TENS (<4 Hz) has been used for impression making.

## Introduction

The field of medicine is constantly evolving, leading to increased life expectancy, hence the number of edentulous patients requiring oral rehabilitation. Complete dentures have been used to rehabilitate edentulous patients for a long time [1]. A complete denture is considered successful only when it attains the prerequisites of stability, support, retention, phonetics, etc. These inherent properties depend on the denture's relation to underlying mucosa, adjoining musculature, and occlusal relation [2,3]. Neuromuscular control pertains to functional forces applied by the patient’s musculature that may affect retention and stability. The cameo surface of a denture should be molded in harmony with peripheral muscles to promote neuromuscular control. Dentures that rest on resorbed ridges require paramount muscular control to enable patients to function [2].

Unsupported lips and cheeks may give an unesthetic appearance, and the arbitrary addition of material to a cameo surface may lead to instability [4]. Hence, various authors recommended that the polished surface of the denture base should be functionally determined [2]. Many techniques have been suggested for impression-making to achieve the optimum limiting structures and the denture's cameo surface [5].

Optimum cameo surface recording of the denture can increase stability in two ways. At first, the action of a few groups of muscles should be allowed to occur without any hindrance by the denture base. It will prevent the probable dislodgement of the prosthesis during function, thereby reducing the chances of compromised stability. Secondly, the operator must acknowledge that certain muscle groups can be used to increase stability during their normal functioning [3].

Transcutaneous electric nerve stimulation (TENS) became popular in clinical dentistry in 1971 when Jankelson recommended it as a method for border molding. Later, Bulbule et al. and Koli et al. discussed using ULF-TENS (Ultra-low frequency TENS) to record the cameo surface of the prosthesis [4,6,7]. The term ULF is given to the frequency of TENS set below 4 Hz. Subsequently, Cooper et al. in 2008 concluded that applying ULF-TENS for 60 minutes allows muscles to attain a physiologic rest position of the jaw due to induced relaxation [8]. Due to the diversity of TENS in prosthodontics, the purpose of the two cases presented here was to provide information on the implications of this technique for rehabilitating clinically challenged, completely edentulous patients.

## Case presentation

Case 1

**Figure 1:**
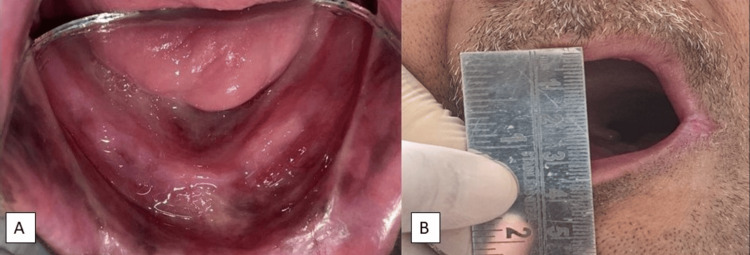
(1A) Intra-oral view depicting partial glossectomy (1B) Extra-oral view showing reduced mouth opening

A 56-year-old male patient presented to the department with a chief complaint of being unable to chew food properly due to missing teeth. History revealed partial glossectomy on the left side due to squamous cell carcinoma of the tongue. The glossectomy procedure occurred around three years ago. After surgical removal of the affected part of the tongue, the patient underwent radiotherapy for six months. The patient had a complete extraction done only a month before he reported it to the Department of Prosthodontics (Figure 1A). The patient had a restricted mouth opening, i.e., 30 mm (maximum mouth opening for Indian males aged 39-70, average inter-commissural width 50-55 mm), and reduced inter-commissural width (Figure 1B).

**Figure 2:**
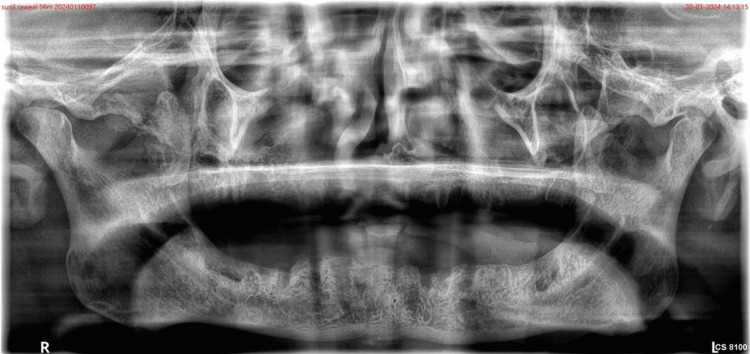
OPG showing reduced bone density OPG: Orthopentomogram

The treatment options available for the patient were an implant-supported removable or fixed prosthesis or a removable complete denture prosthesis. However, due to the inadequate density of mandibular residual bone, a removable complete denture prosthesis was advised for the patient for the maxillary and mandibular edentulous arch (Figure 2). The patient was also not convinced to undergo any surgical procedure. The patient was also having altered speech intelligibility due to partial glossectomy. Hence, a mandibular complete denture with maxillary palatal augmentation prosthesis (PAP) was planned for the patient in which TENS was used for physiologic impressions and the cameo surface.

**Figure 3:**
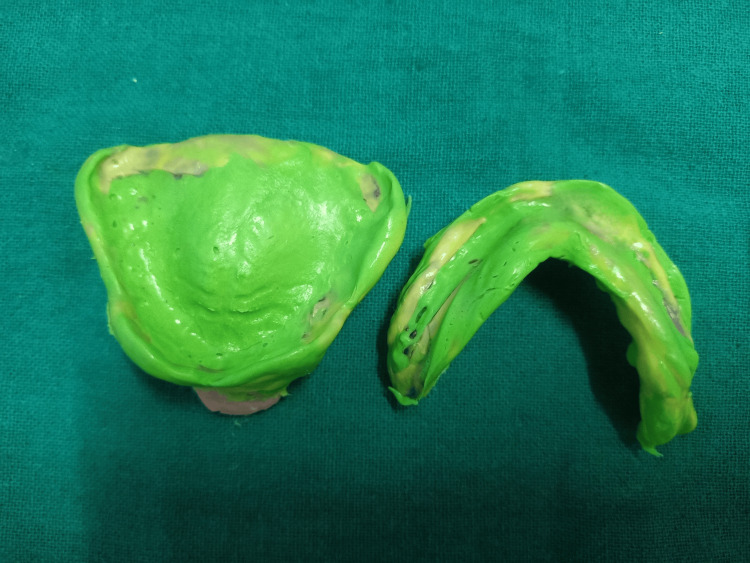
Border molding and wash impressions with PVS PVS: Polyvinyl siloxane

The denture-making procedure was initiated with the primary impressions using impression compound (Y Dent’s, India) followed by one-step border molding and a secondary impression procedure using ULF-TENS (Quadra-TENS, Meditek). TENS was used for definitive impressions with the electrodes placed in the pre-auricular region and the region of the posterior triangle of the neck according to the previous literature by a trained physiotherapist [4]. The unit was set in continuous mode, with the current increasing gradually from 0-4 mA, according to the patient’s tolerance for forty minutes, which allows the physiological contraction of muscles. Subsequently, the putty consistency of polyvinyl siloxane (PVS) impression material (Affinis, Coltene) was adopted on the borders after tray adhesive application (Caulk, Dentsply). Peripheral borders were functionally recorded due to rhythmic contractions of muscles by TENS application followed by wash impression with the light body consistency of PVS material (Affinis, Coltene) (Figure 3).

**Figure 4:**
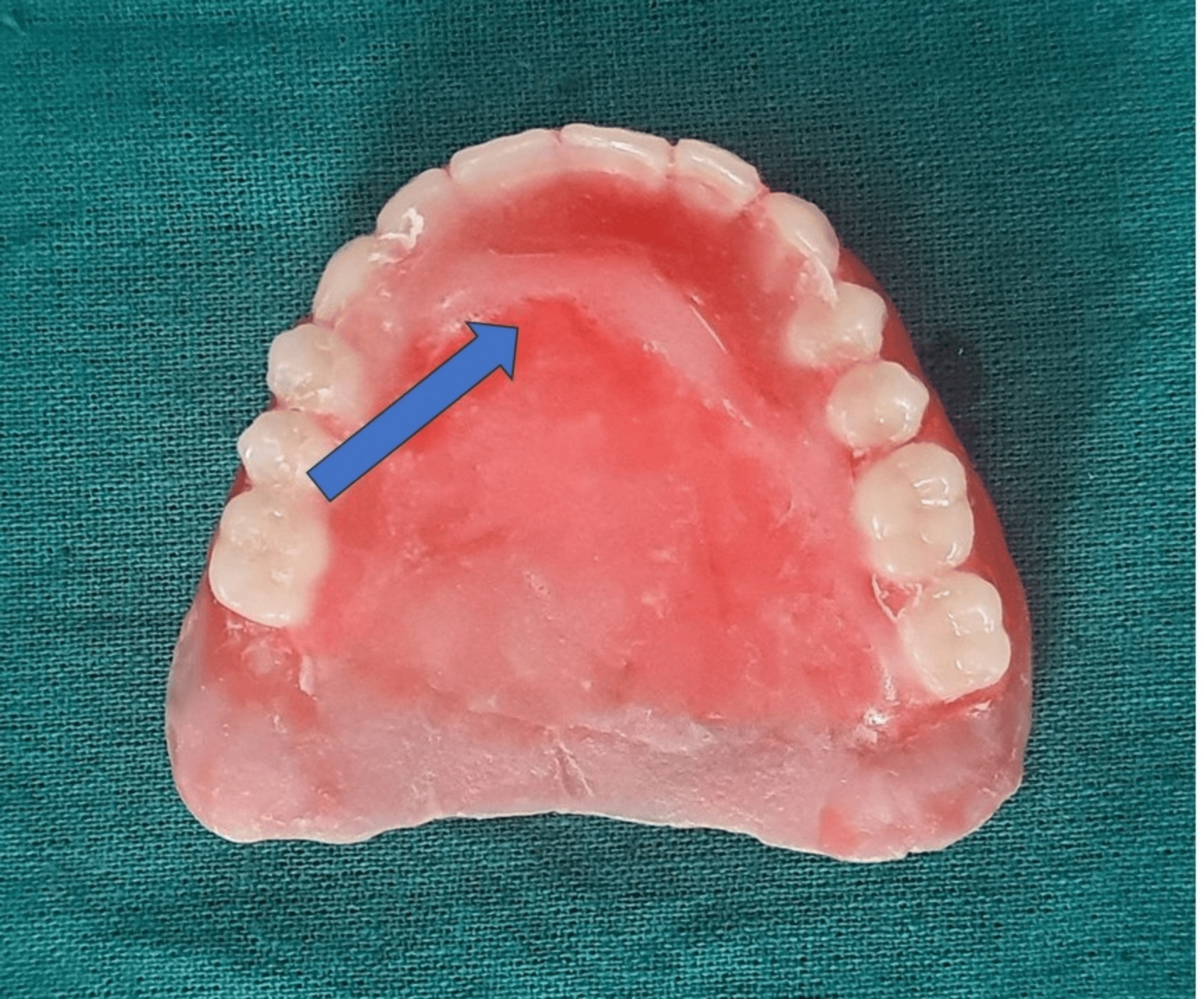
Palatal part recorded in soft tissue conditioner in trial denture base for maxillary PAP PAP: Palatal augmentation prosthesis

Secondary casts were obtained, and denture base and occlusal rims were fabricated, followed by teeth arrangement for try-in. During the try-in, the denture base was lined with tissue conditioning material (Viscogel, Dentsply), and the patient was instructed to pronounce lingual alveolar and linguo-velar sounds. The contact of the tongue with the palate was established by molding tissue conditioner (Figure 4) [9].

**Figure 5:**
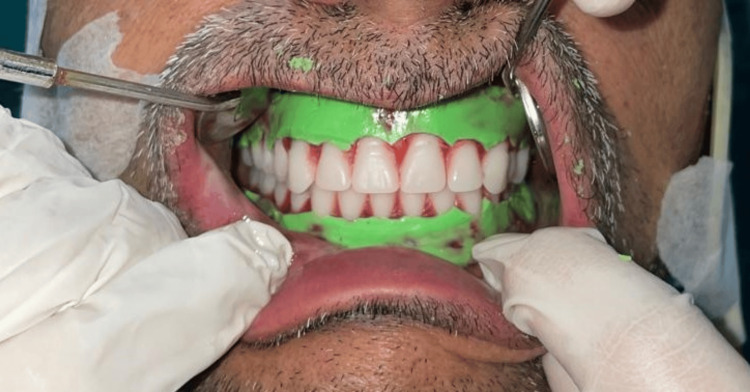
Polished surface recording with light body PVS PVS: Polyvinyl siloxane

After the recording of the palatal part, cameo surface recording was also done using ULF-TENS. ULF-TENS was applied again for 40 minutes to record the muscles in a physiologic state. Light-body consistency of PVS impression material (Affinis, Coltene) was applied on the labial and buccal sides of the maxillary and mandibular denture bases to record the same (Figure 5).

**Figure 6:**
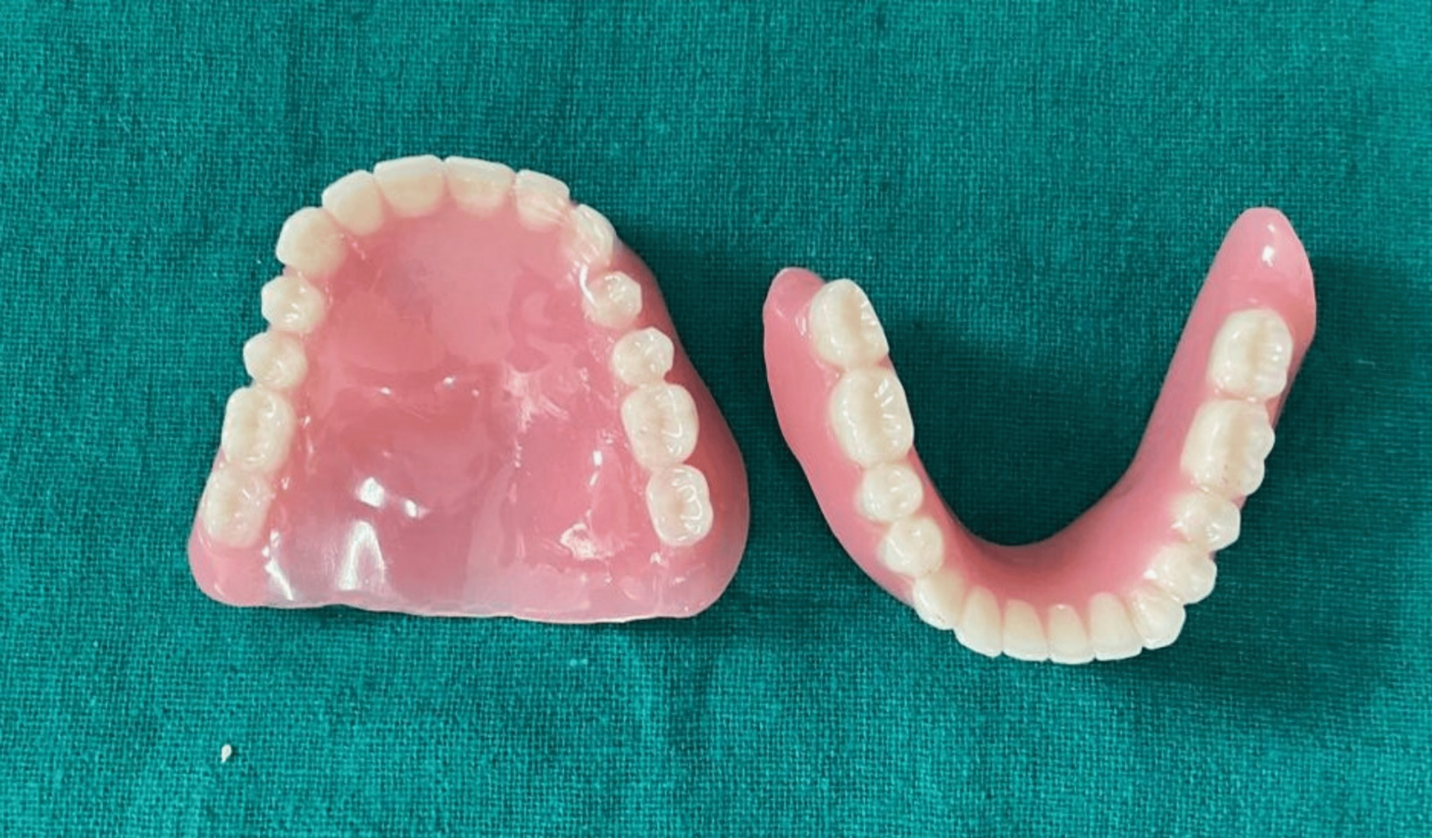
Final complete denture prosthesis

Later, the maxillary PAP and mandibular complete denture were processed in heat cure denture base resin, and denture insertion was carried out (Figure 6).

The patient reported the next day for some minor adjustments in the denture. After two months of recall, the patient was satisfied with the phonetics and deglutition using the prosthesis.

Case 2

**Figure 7:**
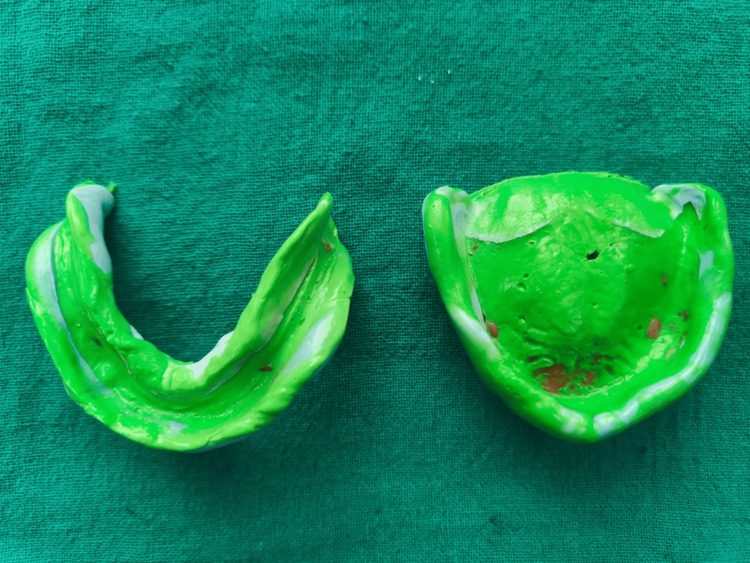
Border molding and final impressions with PVS PVS: Polyvinyl siloxane

A 64-year-old male patient presented to the Department with the chief complaint of difficulty chewing due to ill-fitting dentures. The patient had been wearing a complete denture for the past year, and upon examination, the denture's occlusion and fit were unsatisfactory. The patient was advised an implant-supported prosthesis but being more familiar with removable complete dentures and also due to financial constraints, the patient was not willing to undergo an implant procedure. The patient had an uncomfortable experience during the primary impression procedure when manual manipulation of borders was executed; hence, he opted for the TENS application during definitive impressions. Secondary impressions were made as described in case 1 after ULF-TENS application for 45 minutes in continuous mode and at a current of 3 mA. Rhythmic contractions of muscles in the target area molded the periphery of the impression in a physiological state (Figure 7).

**Figure 8:**
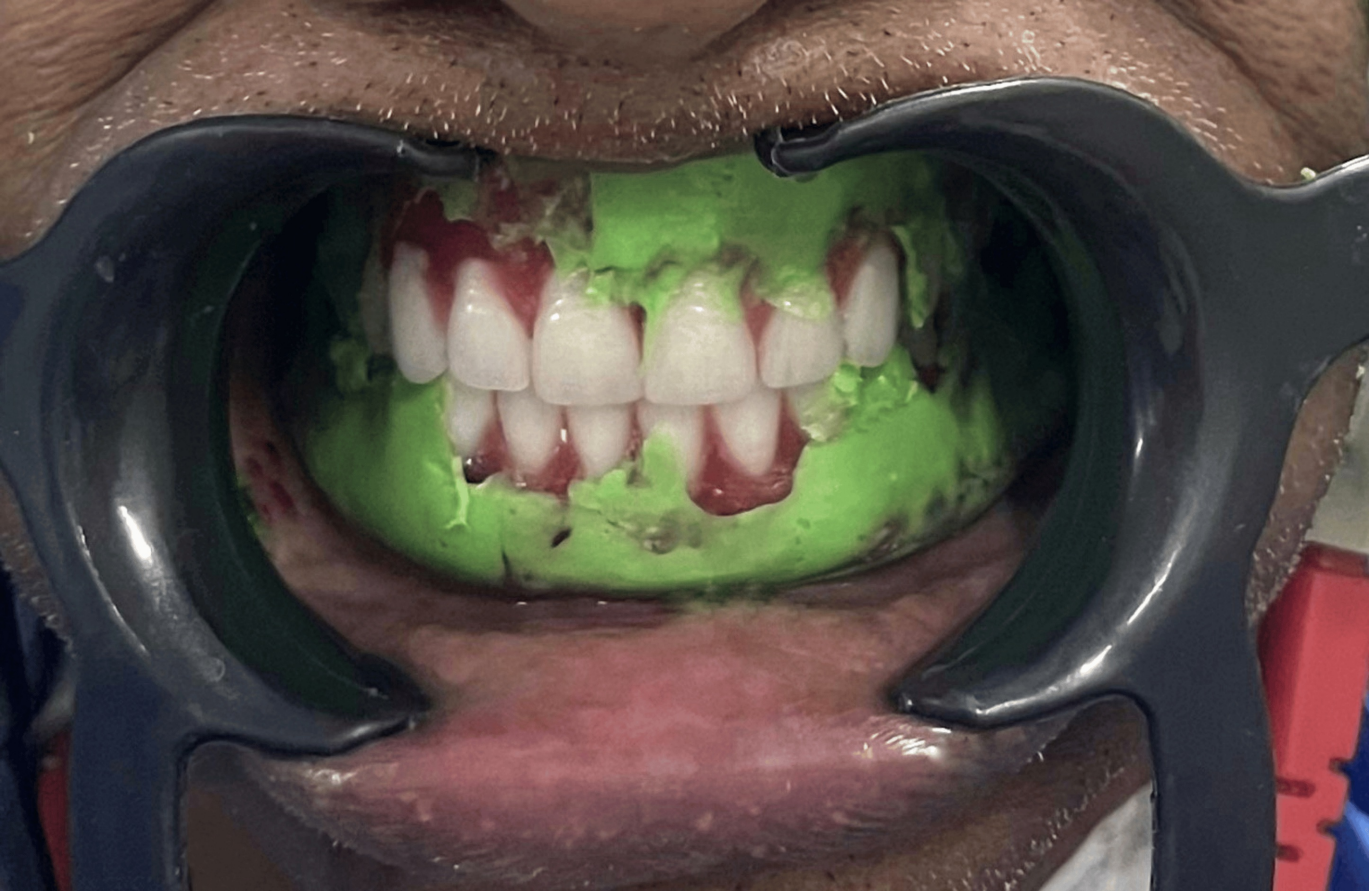
Polished surface recording with light body PVS after application of ULF-TENS PVS: Polyvinyl siloxane; ULF-TENS: Ultra-low frequency transcutaneous electric nerve stimulation

The polished surface was also recorded using the same TENS apparatus and light body PVS impression material (Affinis, Coltene) during the try-in procedure as described in the previous case (Figure 8).

**Figure 9:**
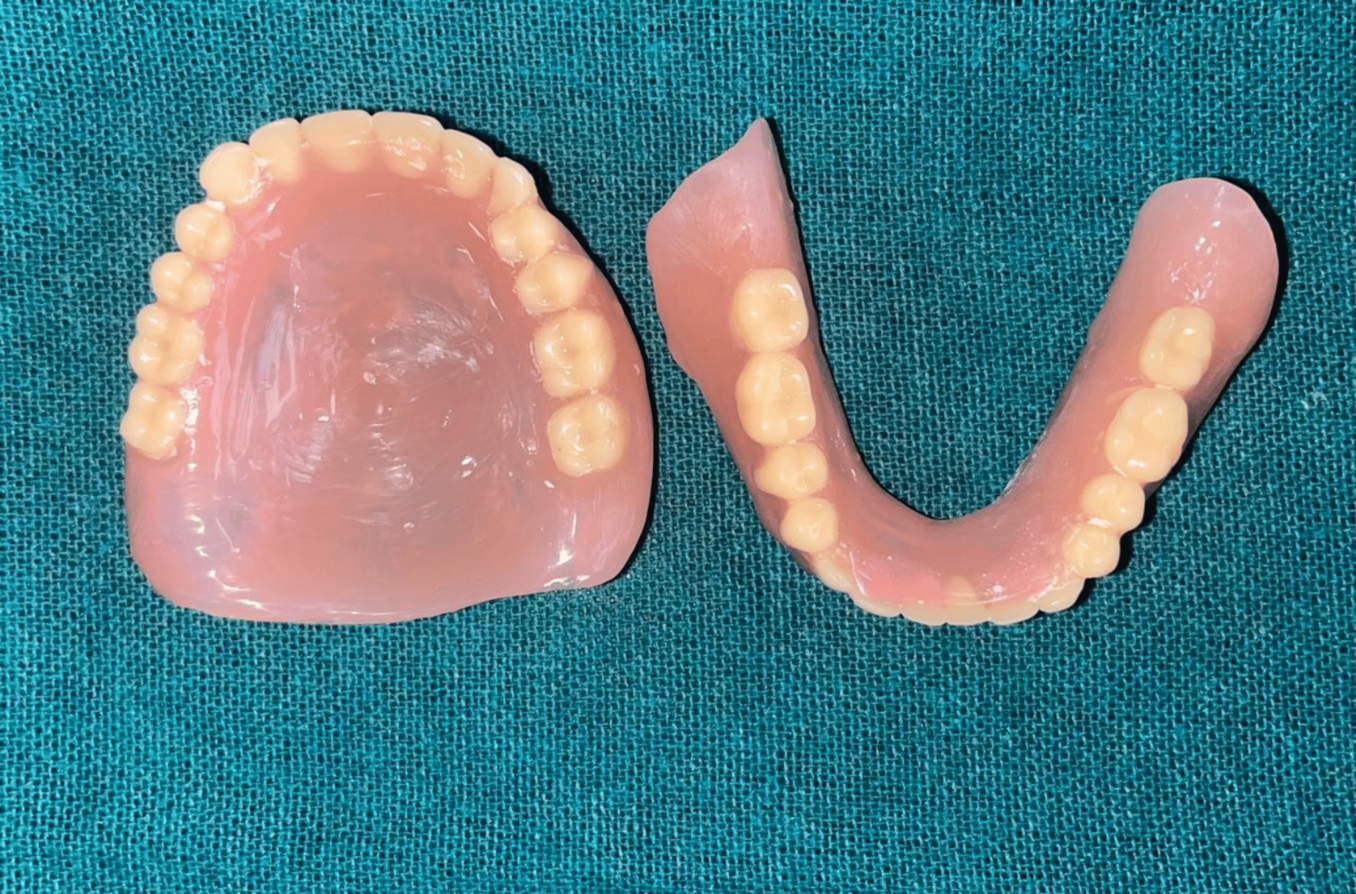
Final prosthesis for insertion

The prosthesis was then processed with heat cure resin material (Dental Products of India, Mumbai, India), and the finished prosthesis was inserted into the patient’s mouth after necessary adjustments (Figure 9).

After one month of recall, the patient was satisfied with the final prosthesis's retention, stability, support, and comfort.

## Discussion

Various methods have been used for the physiological recording of the peripheral borders with the surrounding musculature including TENS. TENS has recently been highlighted because of its clinical applications in various clinical procedures of prosthetic rehabilitation [10,11]. In the presented case 1, the patient had restricted mouth opening, and border molding with the conventional method could have caused discomfort to the patient. In case 2, since the patient had tribulation during manual manipulation of borders for the primary impression, the ULF-TENS was conducted to record the impressions in both cases. Clinically, the application of TENS utilizes various levels of frequencies, i.e., low frequency (frequency <10 Hz), high frequency (>50 Hz), and ULF (<4 Hz) [12]. ULF‑TENS acts on the muscular component in both a dromic and antidromic manner, causing contraction of the 7th and the 5th cranial nerves, resulting in the relaxation of muscles associated with these nerves [13].

The two terminals of each electrode are placed bilaterally, one in the preauricular region, and another in the posterior triangular of the neck. The electrodes placed in front of the tragus stimulate the facial nerve and the mandibular division of the trigeminal nerve. Facial nerve stimulation records the buccinators, levator labii superioris, and orbicularis oris muscles. Stimulation of the mandibular division of the trigeminal nerve brings about activation of the tensor palati muscle. The electrodes placed in the posterior region stimulate the accessory nerve and elicit the action of levator veli palatine, palatoglossus, and palatopharyngeous.

Moreover, activating the vagus nerve causes a generalized relaxing impact on the individual [14]. The current frequency in ULF-TENS was delivered at 2-4 Hz, and its amplitude was gradually raised from 0 to 4 according to their tolerance levels in both patients. TENS may be delivered for 40-60 minutes, though the muscle twitching starts at around 10-12 min [15]. Manual movements were not performed to mold the borders to reduce operator-induced errors in presented cases. However, recording the cameo surface within the physiological limit and in harmony with the surrounding musculature elevates the stability of the denture.

Nanda et al. concluded improved retention and oral health-related quality of life in patients with complete dentures fabricated with TENS therapy [14]. Rajamani et al. and Gowda et al. stated that ULF‐TENS usage leads to a precise recording of the cameo surface by muscle relaxation [16,17]. The presented cases were clinically challenging because, in the first patient, glossectomy led not only to speech problems but also to decreased quality of life. In the second case, conventional border molding was challenging because patient compliance is equally important for proper peripheral molding. The problems of both patients were addressed utilizing the latest techniques in dentistry. The drawbacks of ULF-TENS therapy include that it may cause anxiety in patients due to electrode placement, and it cannot be used in patients with cardiac pacemakers or with systemic diseases which limits electrode placement. Also, it is technique-sensitive and should be implied in the presence of a trained physiotherapist. Consequently, this approach ought to be implemented while contemplating its benefits and drawbacks.

## Conclusions

ULF-TENS is an effective method that helps the physiological adaptation of the peripheral borders and polished surface of the prosthesis to orofacial musculature, especially in a few clinically challenged situations. Moreover, its ability to record physiological final impressions improves quality of life. The use of TENS in patients discussed in this case report notably improved denture performance and patient satisfaction. The limitations of this technique include that it induces anxiety in the patient due to the involvement of electrodes, and a trained physiotherapist is required to conduct the procedure.
